# A164 OPTIMIZING A PROVINCIAL SCREENING PROGRAM FOR DETECTION OF LYNCH SYNDROME

**DOI:** 10.1093/jcag/gwad061.164

**Published:** 2024-02-14

**Authors:** R Winter, Y Liu, J Stone, H Rothenmund, B Chodirker, C Kim, J Klein, H Singh

**Affiliations:** University of Manitoba Max Rady College of Medicine, Winnipeg, MB, Canada; University of Manitoba Max Rady College of Medicine, Winnipeg, MB, Canada; University of Manitoba Max Rady College of Medicine, Winnipeg, MB, Canada; University of Manitoba Max Rady College of Medicine, Winnipeg, MB, Canada; University of Manitoba Max Rady College of Medicine, Winnipeg, MB, Canada; University of Manitoba Max Rady College of Medicine, Winnipeg, MB, Canada; University of Manitoba Max Rady College of Medicine, Winnipeg, MB, Canada; University of Manitoba Max Rady College of Medicine, Winnipeg, MB, Canada

## Abstract

**Background:**

Up to 30% of those with colorectal cancer (CRC) have an affected family member. Lynch syndrome (LS) is the most common inherited condition predisposing patients to CRC. LS is an autosomal dominant condition associated with microsatellite instability (MSI) and mutations in DNA mismatch repair (MMR) genes, and screening CRC cases ≤ age 70 can identify LS in a cost-effective manner. Manitoba launched Canada's first provincial reflex screening program for all CRC cases diagnosed ≤ age 70 using MMR immunohistochemistry (MMR-IHC) in 2017. Our prior 2022 study evaluating the program showed compliance rates of 89.4% for MMR-IHC reflex testing and a 75.8% overall referral rate of screen-positive cases to genetics. However, only 50% were referred directly by pathologists. Several modifications have since been implemented to enhance the program. These included providing feedback to pathologists, creating an “additional testing comment section” on synoptic pathology reports, and regular review of the pathology reports by a pathology assistant. Information materials were developed to encourage genetic testing among relatives.

**Aims:**

Assess whether modifications to Manitoba’s universal screening protocol improved LS patient outcomes and determine any remaining factors to optimize. We specifically aimed to identify current compliance rates for MMR-IHC testing, the overall referral rate of screen-positive cases to genetics, uptake of genetic referrals and genetic testing as well as rate of cascade testing among relatives.

**Methods:**

Data has been obtained by searching the provincial pathology database for “adenocarcinoma” in the colorectal specimen pathology reports. All specimens from 2022 and Q1/Q2 of 2023 were reviewed, meeting the criteria of: a) specimen from colon biopsy/excision containing the term “adenocarcinoma”, b) patient age ≤70 years, and c) missing MMR centralized by the IT/Pathology department.

Additionally, a random sample of cases categorized as “MMR performed” will be audited. We are in the process of reviewing genetic referrals, genetic uptake and the frequency of reminders triggered to each pathologist.

**Results:**

Currently, a total of 1,308 colonic adenocarcinoma specimens (811 from 2022, 497 for 2023) have been reviewed. Appropriate MMR testing was missed in only 1.5% of cases (13/811 =1.6% missed in 2022, 7/497= 1.4% missed in 2023).

**Conclusions:**

Current MMR testing rates for colonic adenocarcinoma specimens within the Manitoba LS screening program have reached a current compliance rate of 98.5%, a substantial improvement from the prior audit (89.4% previously). Further data collection and analysis is ongoing and will also be presented. The reported rates of compliance are the highest that have been reported from any jurisdiction. This study highlights the sustained efforts required to improve detection of LS.

**Funding Agencies:**

None

